# A new *Drosophila* model of cardiac hypertrophy

**Published:** 2013-07

Abnormalities in receptor tyrosine kinase and serine/threonine kinase signalling pathways have been implicated in the development of cardiac hypertrophy, a condition characterised by enlargement of heart muscle cells, which can lead to heart failure and sudden cardiac death. The mechanisms underlying progression to cardiac hypertrophy remain poorly understood. In this study, a group led by Matthew J. Wolf described the first *Drosophila* model of the disorder. The authors report that the affected flies recapitulate the canonical features of human cardiac hypertrophy, including enlargement of cardiomyocytes and a decrease in the volume of the cardiac lumen in response to Raf-mediated molecular signals. This new model, which is free from the complexities associated with mammalian cardiac systems, could provide a platform for the discovery of downstream signalling molecules that contribute to cardiac hypertrophy in humans. **Page 964**

